# Divergent Relationships between Fecal Microbiota and Metabolome following Distinct Antibiotic-Induced Disruptions

**DOI:** 10.1128/mSphere.00005-17

**Published:** 2017-02-08

**Authors:** Jocelyn M. Choo, Tokuwa Kanno, Nur Masirah Mohd Zain, Lex E. X. Leong, Guy C. J. Abell, Julie E. Keeble, Kenneth D. Bruce, A. James Mason, Geraint B. Rogers

**Affiliations:** a Infection and Immunity Theme, South Australia Health and Medical Research Institute, North Terrace, Adelaide, SA, Australia; b King’s College London, Institute of Pharmaceutical Science, London, United Kingdom; c School of Medicine, Flinders University, Bedford Park, Adelaide, SA, Australia; Arizona State University

**Keywords:** beta-lactam, fluoroquinolone, glycopeptide, metabolic activity, microbiota composition

## Abstract

The intestinal microbiome plays an essential role in regulating many aspects of host physiology, and its disruption through antibiotic exposure has been implicated in the development of a range of serious pathologies. The complex metabolic relationships that exist between members of the intestinal microbiota and the potential redundancy in functional pathways mean that an integrative analysis of changes in both structure and function are needed to understand the impact of antibiotic exposure. We used a combination of next-generation sequencing and nuclear magnetic resonance (NMR) metabolomics to characterize the effects of two clinically important antibiotic treatments, ciprofloxacin and vancomycin-imipenem, on the intestinal microbiomes of female C57BL/6 mice. This assessment was performed longitudinally and encompassed both antibiotic challenge and subsequent microbiome reestablishment. Both antibiotic treatments significantly altered the microbiota and metabolite compositions of fecal pellets during challenge and recovery. Spearman’s correlation analysis of microbiota and NMR data revealed that, while some metabolites could be correlated with individual operational taxonomic units (OTUs), frequently multiple OTUs were associated with a significant change in a given metabolite. Furthermore, one metabolite, arginine, can be associated with increases/decreases in different sets of OTUs under differing conditions. Taken together, these findings indicate that reliance on shifts in one data set alone will generate an incomplete picture of the functional effect of antibiotic intervention. A full mechanistic understanding will require knowledge of the baseline microbiota composition, combined with both a comparison and an integration of microbiota, metabolomics, and phenotypic data.

**IMPORTANCE** Despite the fundamental importance of antibiotic therapies to human health, their functional impact on the intestinal microbiome and its subsequent ability to recover are poorly understood. Much research in this area has focused on changes in microbiota composition, despite the interdependency and overlapping functions of many members of the microbial community. These relationships make prediction of the functional impact of microbiota-level changes difficult, while analyses based on the metabolome alone provide relatively little insight into the taxon-level changes that underpin changes in metabolite levels. Here, we used combined microbiota and metabolome profiling to characterize changes associated with clinically important antibiotic combinations with distinct effects on the gut. Correlation analysis of changes in the metabolome and microbiota indicate that a combined approach will be essential for a mechanistic understanding of the functional impact of distinct antibiotic classes.

## Abstract (precis)

Despite the fundamental importance of antibiotic therapies to human health, their functional impact on the intestinal microbiome and its subsequent ability to recover are poorly understood. Much research in this area has focused on changes in microbiota composition, despite the interdependency and overlapping functions of many members of the microbial community. These relationships make prediction of the functional impact of microbiota-level changes difficult, while analyses based on the metabolome alone provide relatively little insight into the taxon-level changes that underpin changes in metabolite levels. Here, we used combined microbiota and metabolome profiling to characterize changes associated with clinically important antibiotic combinations with distinct effects on the gut. Correlation analysis of changes in the metabolome and microbiota indicate that a combined approach will be essential for a mechanistic understanding of the functional impact of distinct antibiotic classes.

## INTRODUCTION

The intestinal microbiome plays an important role in regulating many aspects of host physiology, including glucose and fat metabolism (1–3), systemic immunity (4, 5), and central nervous system function (6, 7). Antibiotic-induced disruption of the intestinal microbiota has been known for some time to be associated with infection, such as by *Clostridium difficile* (8). For example, cefoperazone administration in mice results in the development of an intestinal metabolome that favors *C. difficile* germination and growth (9). In addition, antibiotic-induced perturbation of gut microbiota is being increasingly associated with both the loss of beneficial functions and the gain of microbiota functions that are proinflammatory (10).

The clear importance of the intestinal microbiota to host health has also led to growing concerns about the wider impact of antibiotic interventions on chronic conditions, particularly during human development (11–16). While establishing links between antibiotic exposure and outcome is challenging, large-cohort studies have concluded that the use of certain antibiotics, including vancomycin, cephalosporins, penicillins, and particularly macrolides, can be associated with weight gain (15–18). For example, weight gain in mice has been associated with an increase in *Lactobacillus reuteri* and a decrease in *Escherichia coli* cells as a result of vancomycin administration (19). Changes in short-chain fatty acids (SCFAs) resulting from exposure to vancomycin suggest a functional consequence of antibiotic exposure. A change in a given metabolite therefore may prove to be a more direct link to a particular pathophysiological change than loss or gain of individual taxa (20–22), especially in light of functional redundancy (19, 20, 23). SCFAs are immunomodulatory (21, 22), can interact with nerve cells to stimulate the sympathetic and autonomic nervous system (24, 25), regulate colonic energy metabolism and autophagy (26), and have a range of other important functions (1, 27, 28). Increasing the focus on the metabolome also reduces concern over the impact of variety in baseline gastrointestinal microbiota, which as we have recently shown, differs even in genetically identical mice within the same production facility (29). Studying antibiotic-induced changes by combining the microbiota and metabolome therefore provides a promising route for developing mechanistic insight into their impacts on the microbial community and, in the longer term, on host outcome.

Here, we investigate the relationship between the antibiotic-induced disruption and reestablishment of the intestinal microbiota and the corresponding changes in the fecal metabolome in genetically identical mice. We used two distinct antibiotic treatments, ciprofloxacin and a vancomycin-imipenem combination, that importantly have been shown to differ markedly in their therapeutic impacts on murine microbiota (30), with ciprofloxacin having minimal effects on the anaerobic gut microbiota and vancomycin-imipenem reducing intestinal anaerobes with a broad-spectrum activity (31). Our first aim therefore was to characterize how two substantially different antibiotic regimens have an impact on the same mouse gut microbiota and metabolome. By so doing, we also aimed to determine the additional value resulting from integrating these two approaches in terms of understanding the impact of antibiotic challenge on the murine gut microbial community.

## RESULTS

### Antibiotics alter microbial alpha diversity.

**FIG 1:**
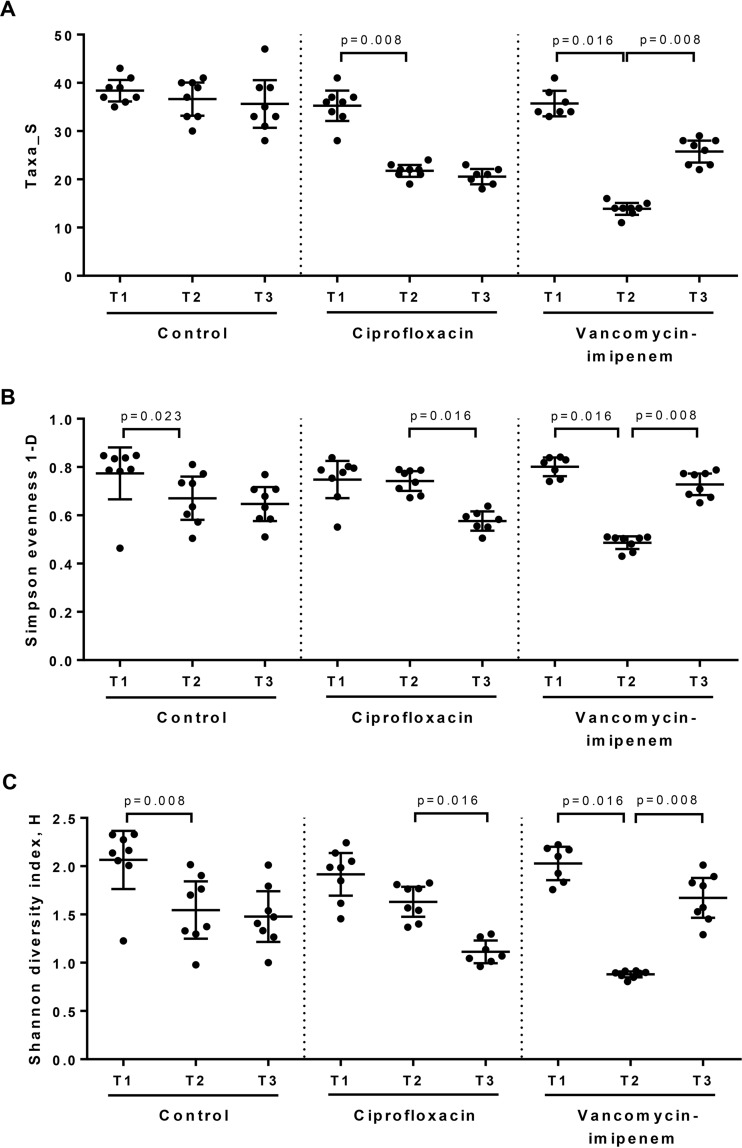
Alpha diversity analysis of fecal microbiota of the control, ciprofloxacin, and vancomycin-imipenem groups. The fecal microbial community of mice was analyzed immediately before treatment (baseline, T1) and after 14 days of antibiotic treatment (T2), as well as at 9 days after antibiotic treatment (T3), to analyze for recovery from antibiotics. Statistical significance between the T1, T2, and T3 time points were analyzed using the Wilcoxon test at a significance level of 0.05.

The fecal microbiota and metabolome were assessed immediately prior to antibiotic treatment (baseline, or time 1 [T1]), after 14 days of antibiotic treatment (during the course of antibiotic treatment, T2), and 9 days following the termination of antibiotics (recovery, T3). Fecal microbiota alpha diversity was assessed across each time point for the control and antibiotic-treated groups. The taxon richness (Taxa_S), evenness (Simpson index), and diversity (Shannon index) of murine fecal microbiota are shown in Fig. 1A to C, respectively. Ciprofloxacin treatment resulted in a significant reduction in taxon richness (*P* = 0.008, Wilcoxon test) but no change in microbiota diversity and evenness. Vancomycin-imipenem treatment resulted in a significant decrease in taxon richness (*P* = 0.016), evenness (*P* = 0.016), and diversity (*P* = 0.016) (Fig. 1).

### Antibiotic treatment resulted in shifts in the fecal microbial composition and structure.

**FIG 2:**
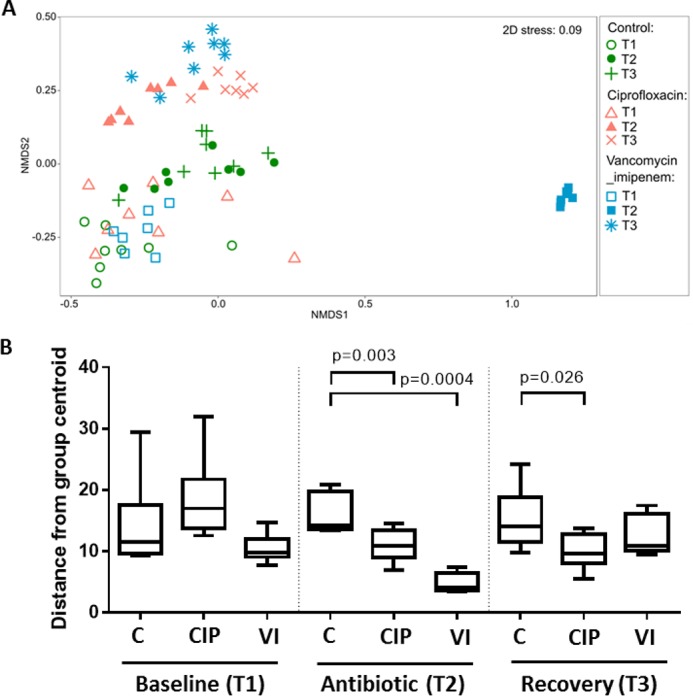
Shifts in the microbial community compositions of the control and antibiotic groups. (A) NMDS plot based on Bray-Curtis distances of the fecal microbiota. Each data point represents a mouse fecal sample from the control, ciprofloxacin, or vancomycin-imipenem group at each time point, with the corresponding labels indicated on the right. (B) The distance from the centroid within each group was analyzed using PERMDISP to determine the homogeneity of dispersion. Statistical significance between the control and antibiotic groups for each time point was analyzed based on the *P* value of the permutation tests at a significance level of 0.05. C, control; CIP, ciprofloxacin; VI, vancomycin-imipenem.

The fecal-microbiota compositions of the control and antibiotic groups were visualized by nonmetric multidimensional scaling (NMDS) based on the Bray-Curtis dissimilarity distances of square root-transformed relative abundances (Fig. 2A; see also the compositional data shown in Fig. S1 in the supplemental material). Permutational multivariate analysis of variance (PERMANOVA) indicated that the gut microbiota compositions between the groups did not differ significantly prior to the antibiotic treatment period (Table S1). Analysis of the distance to a group’s centroid (permutational analysis of the homogeneity of group dispersions [PERMDISP]) also revealed no significant difference in the levels of the homogeneity of dispersions of the microbial community between the control group and the ciprofloxacin {*t* = 1.245, permutation test *P* value [*P*(perm)] = 0.387} or vancomycin-imipenem [*t* = 1.451, *P*(perm) = 0.328] group at baseline (Fig. 2B).

10.1128/mSphere.00005-17.2 FIG S1  Bacterial OTU plots for the control, ciprofloxacin, and vancomycin-imipenem groups at baseline (T1) (A), at the completion of 14 days of antibiotic treatment (T2) (B), and 9 days after cessation of antibiotic treatment (T3) (C). Download FIG S1, PDF file, 0.2 MB. Copyright © 2017 Choo et al. 2017 Choo et al. This content is distributed under the terms of the Creative Commons Attribution 4.0 International license.

10.1128/mSphere.00005-17.6 TABLE S1  PERMANOVA of the microbiota compositions among the control and antibiotic groups accompanied by pairwise comparisons at baseline (T1), at the end of 14 days of treatment (T2), and 9 days after treatment (T3) and the corresponding *Q*^2^ for pairwise OPLS-DA comparisons of metabolomes. Download TABLE S1, XLSX file, 0.01 MB. Copyright © 2017 Choo et al. 2017 Choo et al. This content is distributed under the terms of the Creative Commons Attribution 4.0 International license.

**FIG 3:**
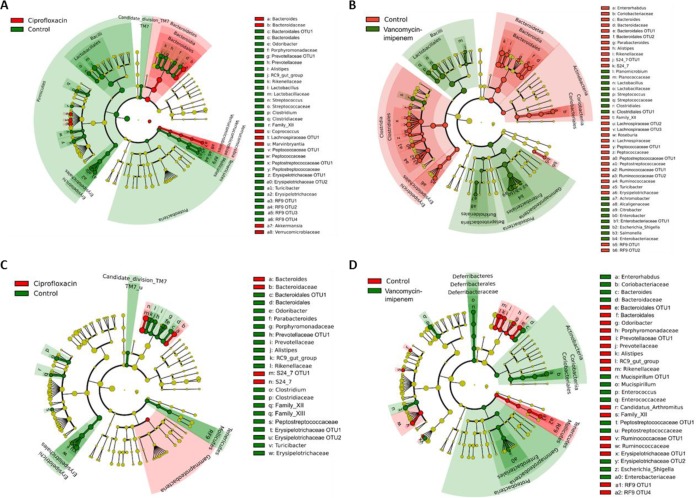
LEfSe comparison analysis between the control and ciprofloxacin or vancomycin-imipenem groups at the end of antibiotic treatment (A or B, respectively) or 9 days after cessation of antibiotic treatment (C or D, respectively). The red or green shading depicts bacterial taxa that were significantly higher in either the control, ciprofloxacin, or vancomycin-imipenem group, as indicated. The yellow circles on the cladogram depict bacterial taxa that were not significantly changed. Selection of discriminative taxa between the control and antibiotic groups were based on an LDA score cutoff of 3.0, and differences in the relative abundances of taxa were statistically determined based on a Mann-Whitney test at a significance level of 0.05.

Within 14 days of antibiotic treatment, the fecal microbial compositions between the control and antibiotic groups were significantly different [*P*(perm) = 0.0001, square root estimated components of variation (ECV) = 41.73, 9,951 permutations] (Table S1). Pairwise comparison indicated that ciprofloxacin significantly altered the microbiota composition from that in the control group [*t* = 3.82, *P*(perm) = 0.0003, 5,069 permutations] (Table S1). Antibiotic-associated effects on the homogeneity of microbiota community dispersion were analyzed using PERMDISP (Fig. 2B), and linear discriminant analysis effect size (LEfSe) analysis was performed to determine bacterial taxa that significantly differed between the control and antibiotic groups (Fig. 3). The distance to the group’s centroid was significantly reduced in the ciprofloxacin group compared to that for controls [*t* = 3.466, *P*(perm) = 0.003] (Fig. 2B), suggesting that microbiota changes resulted from antibiotic selective pressure. LEfSe comparisons indicated that ciprofloxacin resulted in significant decreases in several taxa, including *Odoribacter*, *Alistipes*, *Streptococcus*, *Lactobacillus*, *Clostridium*, *Turicibacter*, and RC9 as well as the *Prevotellaceae* (uncultured) and RF9 families (Fig. 3A), which were completely depleted by ciprofloxacin administration compared to the taxon levels of the control group (Fig. S2). Additionally, increased relative abundances of *Bacteroides*, *Marvinbryantia*, and *Coprococcus* organisms were observed in the ciprofloxacin group.

10.1128/mSphere.00005-17.3 FIG S2  Heatmap analysis of bacterial taxa that significantly differed between the control and ciprofloxacin or vancomycin-imipenem groups at the end of antibiotic treatment and 9 days after cessation of antibiotics, based on LEfSe analysis (LDA score, >3.0). The dendrogram generated represents hierarchical clustering of bacterial taxa based on Bray-Curtis distances and Ward’s method of clustering. The abundance of each bacterial taxon is depicted based on the square root OTU counts. Download FIG S2, PDF file, 1 MB. Copyright © 2017 Choo et al. 2017 Choo et al. This content is distributed under the terms of the Creative Commons Attribution 4.0 International license.

Vancomycin-imipenem also resulted in a significant difference in the fecal microbiota compositions [*t* = 10.43, *P*(perm) = 0.0003, 5,043 permutations] and significantly reduced the distance to the group’s centroid compared to that of the control group [*t* = 8.896, *P*(perm) = 0.0004] (Table S1 and Fig. 2B, respectively). Comparison of the *t* statistic values indicated that these changes were greater than those observed with ciprofloxacin. LEfSe analysis comparing the control and vancomycin-imipenem groups indicated significant depletion of large numbers of taxa, including in members of the *Bacteroidetes* phylum (*Odoribacter*, *Alistipes*, *Bacteroides*, *Parabacteroides*, RC9, S24-7), *Firmicutes* phylum (*Roseburia*, *Candidatus arthromitus*, *Clostridium*, and *Turicibacter*), and *Enterorhabdus*, as well as members of the *Lachnospiraceae*, *Ruminococcaceae*, *Prevotellaceae*, and RF9 families (Fig. 3B). These changes were accompanied by increases in the relative abundance of *Proteobacteria*, including *Enterobacter*, *Escherichia-Shigella*, *Citrobacter*, *Achromobacter*, and *Salmonella*, as well as the genera *Streptococcus*, *Lactobacillus*, and *Planomicrobium*.

### Agent-specific recovery of baseline fecal microbiota following antibiotic treatment.

The degree to which the fecal microbial community had recovered by 9 days following the completion of antibiotic treatment was assessed. Following the recovery period, levels of taxon richness in the ciprofloxacin group remained unchanged; however, microbiota evenness and diversity were significantly reduced compared to levels at antibiotic cessation (*P* = 0.016 in both cases, Wilcoxon test) and to baseline levels (*P* = 0.016 and *P* = 0.031, respectively) (Fig. 1). In addition, a PERMANOVA pairwise comparison indicated that the microbiota composition of the ciprofloxacin group remained significantly different from that of the controls [*t* = 3.04, *P*(perm) = 0.0002, 5,100 permutations] (Table S1). This difference from the microbiota composition at baseline was also significant [*t* = 4.29, *P*(perm) = 0.0003, 5,023 permutations] (Table S2). LEfSe analysis indicated a significantly increased relative abundance of *Bacteroides* organisms and a depletion of *Alistipes* spp., *Odoribacter* spp., and the RC9 gut group in the ciprofloxacin group (Fig. 3C), which failed to be reestablished in the gut microbiota (Fig. S2).

10.1128/mSphere.00005-17.7 TABLE S2  Pairwise comparisons of fecal microbiota compositions and metabolomes at different time points, including at baseline (T1), at the end of 14 days of treatment (T2), and 9 days after treatment (T3) for the control, ciprofloxacin, and vancomycin-imipenem groups. Download TABLE S2, XLSX file, 0.01 MB. Copyright © 2017 Choo et al. 2017 Choo et al. This content is distributed under the terms of the Creative Commons Attribution 4.0 International license.

In the vancomycin-imipenem group, the levels of microbial richness, evenness, and diversity significantly increased 9 days after antibiotic cessation compared to the levels at antibiotic cessation (*P* = 0.008 in all cases, Wilcoxon test) but did not reach baseline levels (*P* = 0.031 in all cases) (Fig. 1). The fecal microbiota composition of the vancomycin-imipenem group also remained significantly different from that of the control group [*t* = 3.45, *P*(perm) = 0.0001, 5,047 permutations] or from the baseline composition [*t* = 6.42, *P*(perm) = 0.0003, 5,045 permutations] (Table S1 and S2, respectively). The extent of the differences between the microbiota compositions in the vancomycin-imipenem group and the control (66.02% mean similarity) was greater than that observed with ciprofloxacin (70.88% mean similarity) based on Bray-Curtis distances, suggesting that recovery of the microbiota was slower with vancomycin-imipenem administration.

In keeping with the posttreatment effects observed with ciprofloxacin, LEfSe analysis revealed that the taxa *Alistipes*, *Odoribacter*, and the RC9 gut group, which were depleted during vancomycin-imipenem treatment, were not restored during the recovery period (Fig. 3D). The relative abundances of several additional taxa, including Family XII and the *Ruminococcaceae*, *Prevotellaceae*, and RF9 families, were also significantly reduced after antibiotic treatment in the vancomycin-imipenem group. The relative abundances of *Escherichia-Shigella* organisms remained elevated, while the relative abundances of *Bacteroides*, *Enterorhabdus*, and *Enterococcus* organisms increased posttreatment.

### Antibiotic-induced dysbiosis results in antibiotic-specific alteration of the fecal metabolome.

**FIG 4:**
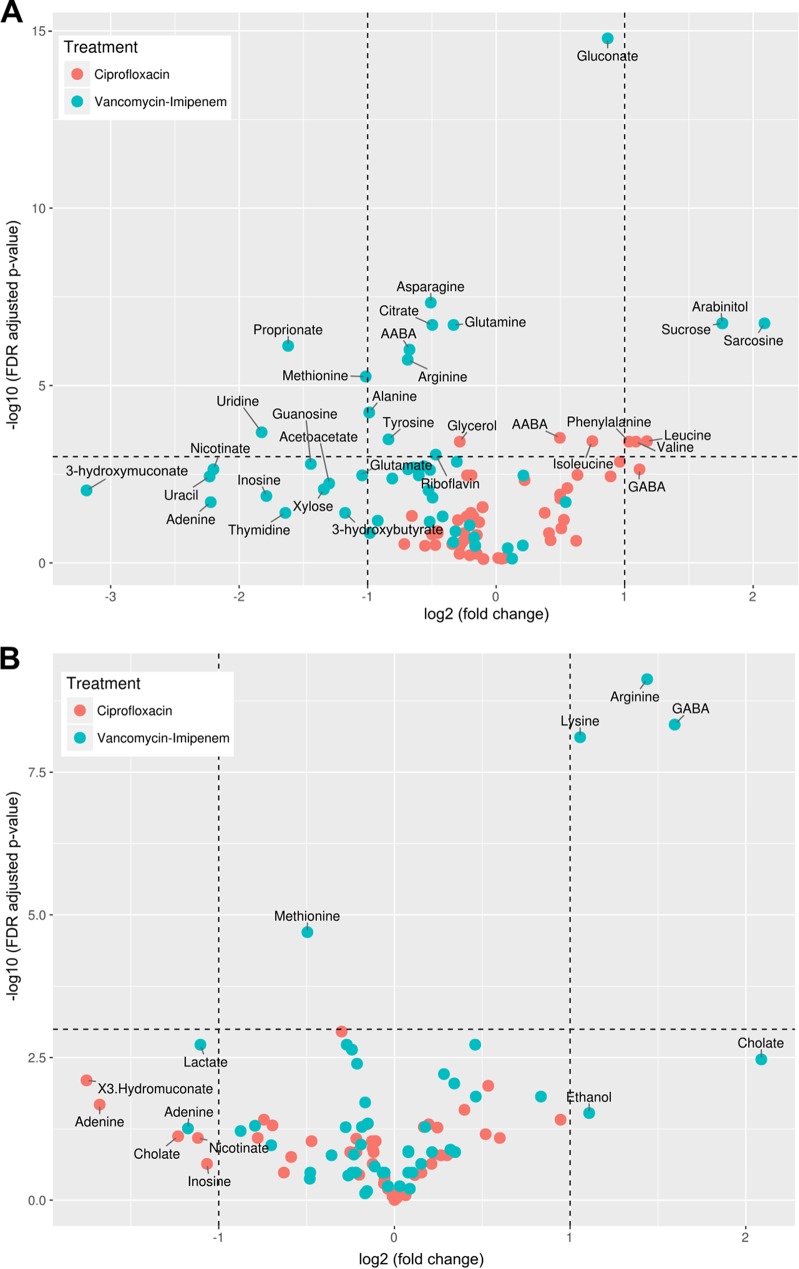
Volcano plot analysis of fecal metabolites altered in the ciprofloxacin or vancomycin-imipenem groups at the end of treatment (A) or 9 days after cessation of antibiotics (B). The *x* and *y* axes of the volcano plot represent the log_2_-fold changes (against the control group) and the corresponding log FDR-adjusted *P* value of all metabolites, respectively. The vertical and horizontal lines separate metabolites that had a 2-fold change and an FDR-adjusted *P* value at 0.05. AABA, α-aminobutyric acid; GABA, γ-aminobutyric acid.

The influence of antibiotic-induced microbiota alterations on the fecal metabolome composition was assessed by NMR spectroscopy. Supervised multivariate analysis based on a cross-validated OPLS-DA (orthogonal projections to latent structures discriminant analysis) model (leave one out) demonstrated that the metabolic profile of the ciprofloxacin group was altered from that of the control group (predictive ability [*Q*^2^] = 0.476). A volcano plot comparing the ciprofloxacin group to the control group indicated that the levels of amino acids, such as valine, leucine, isoleucine, and phenylalanine, as well as α-aminobutyric acid, were significantly increased in the ciprofloxacin-treated group, while levels of the sugar glycerol decreased (Fig. 4A).

The administration of vancomycin-imipenem resulted in a metabolome profile distinct from that the control group (*Q*_2_ = 0.981), and these differences were larger than those observed in the ciprofloxacin group (Fig. 4A). Several metabolites, including the amino acids alanine, methionine, tyrosine, glutamine, arginine, and asparagine, as well as the organic acids citrate, α-aminobutyric acid, and propionate, were found to be lower in the vancomycin-imipenem group than in the controls. The nucleoside uridine was also significantly lower in the treated group than in the controls. Increased levels of sucrose, sarcosine, arabinitol, and gluconate were observed.

### Agent-specific reestablishment of the fecal metabolome following antibiotic dysbiosis.

The OPLS-DA model for the recovery of the fecal metabolome indicated a moderate shift in the metabolite profile of the ciprofloxacin group relative to that of the controls (*Q*^2^ = 0.475), although the corresponding volcano plot analysis indicated that no individual metabolites differed significantly between the two groups (Fig. 4B).

In contrast, the OPLS-DA model describing the metabolic patterns of the vancomycin-imipenem group relative to those of the control group was very strong (*Q*^2^ = 0.872), although only four metabolites were significantly altered when considered individually (Fig. 4B). The vancomycin-imipenem group was again found to have lower levels of methionine but also higher levels of γ-aminobutyric acid, arginine, and lysine than the control group.

### The relationship between altered microbiota composition and changes in the fecal metabolome.

**FIG 5:**
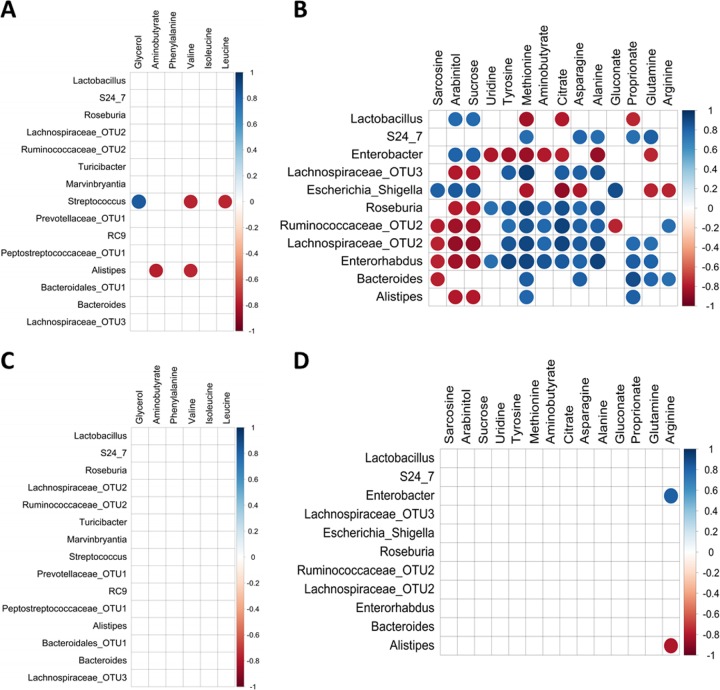
Spearman’s correlation analysis of microbiota and NMR spectra of the control and ciprofloxacin groups (A, C) or the vancomycin-imipenem group (B, D) after 14 days of antibiotic treatment and 9 days after cessation of antibiotics, respectively. Bacterial taxa and metabolites in the correlation matrix were sorted based on the Euclidean distance and Ward’s method of hierarchical clustering. Significant microbiota-metabolite correlations were determined based on an *r* of less than −0.75 or more than 0.75 and an FDR adjusted *P* value of less than 0.01.

The extensive changes in the fecal microbiota and metabolome following exposure to vancomycin-imipenem suggested that these responses can be used to assess microbiota-metabolome associations. The functional correlation between alterations in the microbiota and metabolites was assessed using Spearman’s correlation, based on 11 bacterial taxa (up to a 70% contribution based on a similarity percentage [SIMPER] analysis) and 14 NMR metabolites (fold change significance at a *P* of <0.05) that contributed substantially to the differences between the control and vancomycin-imipenem groups at the end of the treatment (Fig. 5B). The correlation analysis indicated that all 14 metabolites could be correlated with changes in bacterial taxa. The increase in metabolites such as arabinitol and sucrose were positively correlated with increased relative abundances of *Enterobacter*, *Escherichia-Shigella*, and *Lactobacillus* organisms but negatively correlated with *Ruminococcaceae*, *Lachnospiraceae*, and *Enterorhabdus*. Increased levels of sarcosine also resulted in similar microbiota-metabolite relationships, although no correlations were observed with the taxa *Lactobacillus*, *Enterobacter*, and *Roseburia*. Tyrosine, methionine, citrate, asparagine, alanine, and γ-aminobutyric acid shared similar correlation patterns with each other (Fig. 5B). Fewer correlations could be found for the ciprofloxacin-treated group, with only four out of six metabolites correlated with OTU-level changes during treatment (Fig. 5A) and only arginine correlating with OTU during the recovery from vancomycin-imipenem challenge (Fig. 5D).

The potential for changes in the intestinal microbiota to explain associated changes in the metabolome were investigated using PICRUSt. This approach was used to predict shifts in the prevalences of genes that encode metabolic traits, based on changes in the relative abundances of detected taxa. Metabolic pathways predicted to alter significantly as a result of changes in microbiota composition are shown for antibiotic treatment and at recovery periods for ciprofloxacin and vancomycin-imipenem (Fig. S3A and B and S4A and B, respectively). While such projections do not take into consideration cross-metabolism between taxa or levels of gene transcription, these results were consistent in many cases with observed microbe-metabolite correlations. For example, treatment with vancomycin-imipenem resulted in a decrease in the abundance of metabolites involved in the alanine, aspartate, and glutamate metabolic pathways. In keeping with these observed changes, predicted shifts in the metagenome included a decrease in the carriage of genes that encode related enzymes. Decreases in aspartate aminotransferase (an enzyme involved in the conversion of l-aspartate to oxaloacetate and glutamine), aspartate 4-decarboxylase (which directly converts l-aspartate to l-alanine), and alanine dehydrogenase (which is involved in converting pyruvate to l-alanine) were predicted, while genes encoding alanine-synthesizing transaminase (involved in the interconversion between pyruvate and l-alanine) increased in prevalence. For enzymes that were involved in the conversion of succinate semialdehyde to either glutamate or succinate, significant reductions in 4-aminobutyrate aminotransferase/(*S*)-3-amino-2-methylpropionate transaminase (involved in the conversion to glutamate) were observed, while an increase in succinate-semialdehyde dehydrogenase (NADP^+^) (involved in the conversion to succinate) was observed. Decreases were seen in 1-pyrroline-5-carboxylate dehydrogenase (involved in the production of l-glutamate) and in glutamate synthase and glutaminase (involved in the direct conversion of glutamine to glutamate), and an increase in the prevalence of alanine-synthesizing transaminase was also predicted.

10.1128/mSphere.00005-17.4 FIG S3  Predicted changes in bacterial contributions to the fecal metabolome as a result of ciprofloxacin treatment (A) and during recovery after ciprofloxacin treatment (B), estimated based on changes in the observed taxon relative abundances using PICRUSt. Mean proportions of pathways assigned to control and treatment groups (percentages) and Welch's *t* test with Benjamini-Hochberg false-discovery rate-corrected significance are shown. Download FIG S3, PDF file, 0.2 MB. Copyright © 2017 Choo et al. 2017 Choo et al. This content is distributed under the terms of the Creative Commons Attribution 4.0 International license.

10.1128/mSphere.00005-17.5 FIG S4  Predicted changes in bacterial contributions to the fecal metabolome as a result of vancomycin-imipenem treatment (A) and during recovery after vancomycin-imipenem treatment (B), estimated based on changes in the observed taxon relative abundances using PICRUSt. Mean proportions of pathways assigned to control and treatment groups (percentages) and Welch's *t* test with Benjamini-Hochberg false-discovery rate-corrected significance are shown. Download FIG S4, PDF file, 0.3 MB. Copyright © 2017 Choo et al. 2017 Choo et al. This content is distributed under the terms of the Creative Commons Attribution 4.0 International license.

PICRUSt-derived predictions of changes in gene prevalence also included those involved in arginine biosynthesis and tyrosine metabolism, pathways which showed significant changes in NMR-derived metabolome profiles. For example, there was a decrease in the prevalence of acetylglutamate/acetylaminoadipate kinase, *N*-acetyl-gamma-glutamyl-phosphate reductase, and acetylornithine aminotransferase, each of which is involved in the conversion from *N*-acetylglutamate to *N*-acetylornithine (a substrate for arginine synthesis). Finally, an increase in genes encoding 4-hydroxyphenylpyruvate dioxygenase, an enzyme that converts 4-hydroxyphenylpyruvate (a substrate for tyrosine metabolism) to homogentisate, were consistent with the observed decrease in tyrosine levels. Furthermore, in keeping with the observed increase in the abundance of arabinitol in the vancomycin-imipenem-treated mice, there was a significant decrease in the predicted prevalence of genes encoding xylulokinases, which are involved in its catabolism.

Changes in microbiota and metabolome composition were less marked in mice treated with ciprofloxacin. However, a predicted decrease in the carriage of acyl coenzyme A (acyl-CoA) dehydrogenase was consistent with the observed increase in the abundance of valine, leucine, and isoleucine.

## DISCUSSION

The gut microbiota is critical for human health (32–34), with a number of essential metabolites derived exclusively through the activities of these intestinal microbes (30, 35, 36). The gut microbiota also produces of a wide range of compounds that modulate host physiology (37), including immune regulation, metabolic control (32, 38), central nervous system function (39), and xenobiotic metabolism (40). Analysis of antibiotic-induced disturbances in the gut microbiota and its corresponding metabolome can therefore provide insight into both acute and chronic effects of antibiotics on the host and may yield a functional understanding of the development of associated conditions (41). A number of previous studies have used C57BL/6 mice to investigate links between antibiotic-induced changes in intestinal microbiota composition and host physiology (42–45), while assessment of metabolomic changes have been limited to other genetic backgrounds (10, 46).

The present study highlights a number of functional effects on the mouse gut microbiome and metabolome which warrant further investigation and are potential starting points for a mechanistic understanding of the role of various antibiotic regimens in, e.g., gut inflammation and/or obesity. More importantly, the present study reveals substantial variation in dynamic relationships between individual taxa, groups of taxa, and metabolites at different stages of antibiotic intervention. The following examples indicate that while some interesting information may be determined by examining either microbiota or the metabolome, the most informative mechanistic investigations will involve a combined assessment of the relationships between microbiota and the metabolome for a given phenotypic trait.

In a first example, vancomycin-imipenem treatment resulted in substantial changes in microbiota composition, including the depletion of members of the *Ruminococcaceae* and *Lachnospiraceae* families and increases in the relative abundances of *Enterobacteriaceae* and *Lactobacillus* spp., with concomitant changes in the levels of a large number of metabolites. The potential for microbiota-level changes to influence disease risk by altering the composition of the metabolome are well illustrated by such changes. For example, arabinitol is poorly absorbed in the intestine, and its removal is based on its conversion to pentose sugars by taxa, including *Lachnospiraceae* and *Ruminococcaceae* (47). Further, the prevalence of genes encoding arabinitol-catabolic enzymes was predicted to be significantly reduced based on changes in relative taxon abundances. We observed a negative correlation between the relative abundances of *Lachnospiraceae* and *Ruminococcaceae* and fecal arabinitol levels. Increased levels of arabinitol and sugars, such as sucrose, that were also increased during vancomycin-imipenem treatment have been associated with susceptibility to *C. difficile* infection in mice and might act as a growth substrate (9). Further, the increased relative abundance of *Enterobacteriaceae* that accompanied decreases in the abundances of *Lachnospiraceae* and *Ruminococcaceae* are strongly associated with gut inflammation (48). These changes correlated with decreased glutamine levels, which has anti-inflammatory effects potentially through its role in the maintenance of tissue permeability and its inhibitory action on NF-kB activation and p38 mitogen-activated protein kinase (MAPK) pathways based on animal and human intestinal studies (49, 50).

In contrast, the functional relationships between bacterial taxa and, e.g., arginine are more complex. The reestablishment of microbiota/metabolome composition 9 days after cessation of vancomycin-imipenem treatment was characterized by significant increases in arginine. These increases were correlated with increases in *Enterobacter* spp. and decreases in *Alistipes* spp. Again, the prevalence of genes encoding enzymes in the arginine biosynthesis pathway were predicted to be significantly increased, based on taxon relative abundances. Arginine plays an important role as a precursor for immune-modulatory compounds (51–53), and the functional significance of antibiotic-induced metabolite changes in arginine may therefore be explored in future studies. Changes in arginine were also observed, however, immediately at the end of the 14-day treatment with vancomycin-imipenem. Here a modest but significant reduction in arginine was instead correlated with depletion of *Ruminococcaceae* and *Bacteroides* spp. and increases in *Escherichia-Shigella* spp., and there was no correlation with either *Enterobacter* or *Alistipes* spp. Arginine levels in the gut may therefore respond to changes in the prevalence of multiple OTUs, and there may be considerable functional redundancy. As such, given that we have previously observed functional divergence in gastrointestinal microbiotas even in genetically identical mice that originate in the same production facility (29), it may be inaccurate to identify individual OTUs as responsible for a particular functional impact without considering the baseline microbiota composition or without supporting metabolomic data.

In a second example, the levels of severity of antibiotic impact can be substantially different when assessed by next-generation sequencing and NMR metabolomics. In keeping with studies of humans (54–56), the administration of ciprofloxacin also substantially reduced microbial richness and evenness. Here, normal microbiota composition was not restored in the mice within the 9 days after antibiotic treatment, a process that can take up to a month in humans (54, 55). Changes in microbiota compositions were accompanied by changes in levels of fecal metabolites. However, while levels of metabolites such as valine, leucine, isoleucine, and phenylalanine significantly increased, they returned to the levels observed for the control group in the 9 days following antibiotics. Notably, supplementation of these amino acids has been shown to promote insulin resistance in humans (57) and rats (58) and to increase the risk of type 2 diabetes (59).

We observed many instances of significant correlation between changes in bacterial taxa and metabolites. However, disparities in the dynamics of microbiota and metabolome disruption in response to antibiotics, and subsequent restoration of baseline levels, highlight the importance of assessing both systems. For example, several bacterial taxa, particularly members of the *Bacteroidetes* phylum, were substantially depleted by ciprofloxacin treatment and did not recover during the 9 days after cessation of the antibiotics. However, ciprofloxacin-driven disruption of the metabolome was less substantial, with its composition following the recovery period broadly in keeping with the baseline. Such discrepancies between the microbiota and metabolome-level antibiotic effects are a further indication of functional redundancy in the intestinal microbiome, the need to examine both composition and function in order to fully characterize the impacts of antibiotic therapy, and the potential influence of antibiotics on host physiology.

Our study had a number of limitations that must be taken into consideration. We examined the impact of antibiotic exposure on the fecal microbiota and correlated this with changes in the compositions of the fecal metabolome. However, establishing direct links between bacterial taxon relative abundances and levels of specific metabolites is extremely challenging. Cross-metabolism between species means that many different taxa can contribute to particular pathways, with functional redundancy between phylogenetically distant populations potentially offsetting reductions in other populations. Furthermore, antibiotics may influence aspects of host physiology, such as appetite, or the production of mucins and immunoglobulins, providing a host-mediated path to metabolome alterations.

Identifying links between antibiotic exposure, microbiota compositional change, an altered fecal metabolome, and disrupted host physiology is extremely challenging. However, the combined use of the sequencing-based bacterial community analysis and metabolomic profiling described here represents an important strategy in elucidating such relationships.

## MATERIALS AND METHODS

### Murine fecal samples.

Feces were collected from female C57BL/6 mice at Charles River, Inc., United Kingdom Ltd. (Margate, United Kingdom) under a commercial license, and all mice were maintained and used in accordance with the Animal Scientific Procedures Act (60) and amendment regulations of 2012 (61). All mice were initially housed in one barrier room within the facility and then transferred to an isolator cage on commencement of antibiotic treatment, where they were divided into three groups of 8 mice each (4 mice per cage). The three groups represented a control group (no treatment) and two antibiotic treatment groups (either vancomycin-imipenem or ciprofloxacin). Six-week-old mice were placed on the same diet (a VRF1 diet, SDS), and antibiotics were dosed at 50 mg/kg of body weight/day delivered in drinking water. Fresh medicated solutions were prepared weekly and stored at 4°C. Water was changed twice weekly, and mice were observed closely for any changes in hydration or for adverse effects; mice were weighed once a week throughout the study. No significant differences in mass were observed between groups during the study, with mice in the control, ciprofloxacin, and vancomycin-imipenem groups attaining masses of 19.26 ± 1.38 g, 19.45 ± 1.11 g, and 19.03 ± 0.81 g, respectively (weights for individual animals are shown in Table S3 in the supplemental material). One fecal pellet was taken from each mouse at three time points: T1, prior to commencement of antibiotic treatment, T2, at termination of treatment after 14 days on antibiotics, and T3, 9 days after antibiotic treatment ceased. After collection, pellets were placed into separate, sterile collection tubes and frozen at −80°C prior to analysis.

10.1128/mSphere.00005-17.8 TABLE S3  Weights of individual animals in the control and antibiotic treatment groups at each of the three assessment points. Download TABLE S3, XLSX file, 0.01 MB. Copyright © 2017 Choo et al. 2017 Choo et al. This content is distributed under the terms of the Creative Commons Attribution 4.0 International license.

### Sample processing.

Known masses of mouse feces were immersed in 1 ml of cold (4°C) 1× sterile phosphate-buffered saline (PBS; pH 7.4) (Thermo Fisher Scientific, United Kingdom) and centrifuged at 13,000 × *g* for 10 min to form a pellet for DNA extraction. The supernatant was transferred to a fresh microcentrifuge tube for metabolomics study. DNA extraction was performed using a Mo Bio PowerLyzer PowerSoil DNA isolation kit (Mo Bio Laboratories, Carlsbad, CA, USA), as previously described (62).

### 16S rRNA gene amplicon sequencing.

Amplicons of the v4 hypervariable region of the bacterial 16S rRNA gene were generated from DNA extracts, as described previously (62). Briefly, amplicons were generated from 25 PCR cycles, and indexes were attached to the amplicon with 8 PCR cycles. Sequencing was performed on an Illumina MiSeq platform at the David R. Gunn Genomics Facility, South Australian Health and Medical Research Institute. Full details are provided in the supplemental material. Paired-end 16S rRNA gene sequence reads were analyzed with the Quantitative Insights into Microbial Ecology (QIIME) software (v1.8.0) (63) using a bioinformatics pipeline described previously (64). Bar-coded forward and reverse sequencing reads were quality filtered and merged using Paired-End reAd mergeR (PEAR, v0.9.6) (65). Chimeras were detected and filtered from the paired-end reads using USEARCH (v6.1) (66) in a comparison with representative sequences from the Greengenes database (v13.8) that clustered with 97% similarity (67). Operational taxonomic units (OTUs) were assigned to the reads using an open-reference approach with the UCLUST algorithm (v1.2.22q) in a comparison with sequences in SILVA database release 111 (July 2012) (68) that clustered at 97% identity. During the OTU assignment, sequences preclustered at 80% similarity to the reference sequence prior to *de novo* clustering. All samples were subsampled to 6,250 reads based on the lowest read depth.

### ^1^H NMR metabolomics.

As described in the paragraphs above, supernatants were obtained from the mouse feces resuspended in cold PBS. Supernatants were frozen by immersion in liquid nitrogen, lyophilized at −58°C overnight, and resuspended in 500 µl D_2_O. ^1^H NMR spectra were acquired under automation at 298 K and 700 MHz on a Bruker Avance II 700 NMR spectrometer (Bruker BioSpin, Coventry, United Kingdom) equipped with a 5-mm helium-cooled quadruple resonance cryoprobe and a cooled SampleJet sample changer. The temperature was allowed to stabilize for 3 min after insertion into the magnet. Tuning, matching, and shimming was performed for each sample, and the ^1^H pulse length was calibrated on each sample and was typically around 12 μs. One-dimensional (1D) Carr-Purcell-Meiboom-Gill presaturation (CPMG-presat) (cpmgpr1d) experiments were acquired with 128 transients, a spectral width of 20.5 ppm, 64,000 data points, a mixing time of 10 ms, a relaxation delay of 4 s, and a total echo delay of 78.7 ms. 1D nuclear Overhauser effect spectroscopy (NOESY)-presat (noesygppr1d) data were also acquired but with 32 transients. Free-induction decays were multiplied with an exponential function (line broadening of 0.3 Hz), Fourier transformed, and calibrated to a 2,2,3,3,-D4-3-(trimethylsilyl) propionic acid (TSP) reference signal at 0.0 ppm. Phase correction was performed manually, and automatic baseline correction was applied. To help in the assignment of metabolite resonances, 2D J-resolved (jresgpprqf) and correlation spectroscopy (COSY) (cosygpprqf) spectra were acquired for a subset of samples. In 2D J-resolved spectra, 64 transients of 8,000 data points were acquired for each of 40 increments, with a relaxation delay of 2 s, during which the solvent signal was suppressed by presaturation. Spectral widths of 16 ppm and 78 Hz were used. In 2D COSY spectra, 8 transients of 4,000 data points were acquired for each of 400 increments, with a relaxation delay of 2 s, during which the solvent signal was suppressed by presaturation. A spectral width of 16 ppm was used in each dimension.

Preprocessing and OPLS-DA (orthogonal projections to latent structures discriminant analysis) were carried out with both MVAPACK (69) and software that was developed in our laboratory for a previous study (70) using the Python programing language with NumPy and SciPy for calculations and matplotlib for visualization. The nonlinear iterative partial least-squares (NIPALS) algorithm (71) was used for OPLS-DA. For integrated microbiome and metabolomic analysis, consensus OPLS were created using the K-OPLS R package (72).

Regions above 8.5 ppm and below 0.5 ppm were excluded because of noise content. The water peak and TSP reference signal were also excluded. Spectra further aligned with the icoshift (73) algorithm and bucketed with the optimized bucketing algorithm (74), using a 0.005-ppm minimum bin size, leaving 1,652 data points per spectrum. These spectra were subjected to probabilistic quotient normalization (PQN) and Pareto scaled (75).

### Data analysis and statistics.

Microbial data were analyzed for alpha diversity measures (taxon richness, *S*; Shannon-Wiener index, *H*; Simpson diversity index, 1 to *D*) of the microbial community using PAST (v3.04) (76). Operational taxonomic unit (OTU) relative abundance was imported into the Primer-E software (v.6; Primer-E Ltd., Plymouth, United Kingdom) for beta diversity analysis. Bray-Curtis similarities were calculated based on the square root-transformed OTU relative abundances and were used in the nonmetric multidimensional scaling (NMDS) ordination plot. The permutational analysis of variance (PERMANOVA) model was used for testing the null hypothesis of no difference between the compared groups (54), based on the parameters’ permutation of residuals under a reduced model and a type III sum of squares. PERMDISP was used to assess the dispersion of the microbial community within the groups (54). Microbial differences between groups at the taxonomic level were tested for statistical significance based on the Kruskal-Wallis and Mann-Whitney tests using GraphPad Prism 6 (GraphPad Software, Inc., La Jolla, USA). The LEfSe algorithm was performed using the Galaxy application tool (http://huttenhower.sph.harvard.edu/galaxy/) with a linear discriminant analysis cutoff score of 3.0 and a *P* of <0.05 for statistical significance (55). Functional predictions of microbial community were performed using PICRUSt on closed-reference OTUs with 97% identity based on the Greengenes database (v13.5) (56). The OTUs were normalized on PICRUSt and used for the prediction of KEGG orthologs (KOs). The predicted metagenome was statistically analyzed on STAMP using Welch’s *t* test with the Benjamini-Hochberg correction for the false-discovery rate (FDR) and filtered to retain features with an effect size (ratio of proportions) of greater than two (77). A heatmap was generated using the ggplots2 package (v2.0.0), and a dendrogram of bacterial taxa was generated based on the Bray-Curtis distances and hierarchical clustering performed using Ward’s method (78, 79). Two and three mice, respectively, were excluded from the microbiota and metabolome analysis, as samples from them failed quality control thresholds.

For analysis of the metabolome data, cross-validation was performed in the same manner for OPLS-DA and consensus OPLS-DA. Seventy-five percent of the samples were used as a training set, and the remaining 25% were used as a test set, ensuring that the number of samples in the test set was proportional to the total number of samples from each class and that at least one sample from each class was present in the test set. To choose the number of components for the model, a leave-one-out cross-validation step was carried out on the samples in the training set, and the F1 was used to choose the number of components, with the additional constraint that a maximum of 8 components was used. A double cross-validation was repeated 2,000 times for each group with randomly chosen samples in the training and test sets to prevent bias due to the choice of training or test set.

This procedure was repeated with randomly generated class assignments to provide a reference value for *Q*^2^. The chosen number of components minus one was then used as an OPLS filter, and a PLS-DA with two components was carried out on the filtered data to yield one predictive and one orthogonal component. In the back-scaled loadings analysis, peaks that allow the models to distinguish between classes were assigned by comparing chemical-shift values and multiplicities from J-resolved NMR spectra to values from the BMRB (80), HMDB (81), and Chenomx software. Volcano plots were generated in R. Five samples were excluded from the metabolome analysis, as these samples failed quality control thresholds. Microbiota and metabolome associations based on Spearman’s correlation were performed in R using the corrplot package. Bacterial taxa and metabolites were sorted according to hierarchical clustering based on Euclidean distances, and Ward’s method was used to perform cluster analysis.[Supplementary-material textS1]

10.1128/mSphere.00005-17.1 TEXT S1  16S rRNA gene amplicon sequencing. Download TEXT S1, PDF file, 0.02 MB. Copyright © 2017 Choo et al. 2017 Choo et al. This content is distributed under the terms of the Creative Commons Attribution 4.0 International license.

### Accession number(s).

Microbial sequencing data have been deposited in the Sequence Read Archive (SRA) database under GenBank accession number SRP096906. Metabolite data have been deposited in the MetaboLights database under accession number MTBLS422.
