# Supplementary figures and images for: Divergent Relationships between Fecal Microbiota and Metabolome following Distinct Antibiotic-Induced Disruptions
Source: mSphere. 2017 Feb 8;2(1):e00005-17. doi: 10.1128/mSphere.00005-17 (PMC5299068; doi:10.1128/mSphere.00005-17)

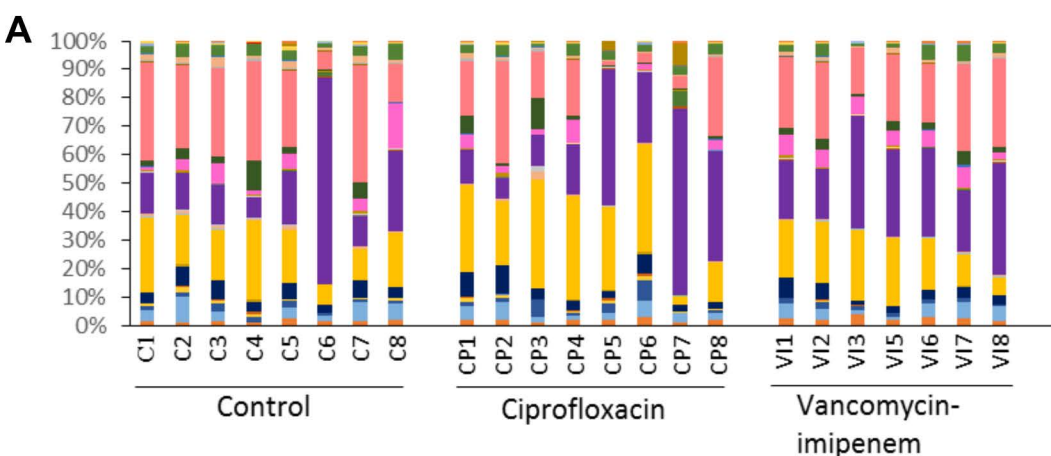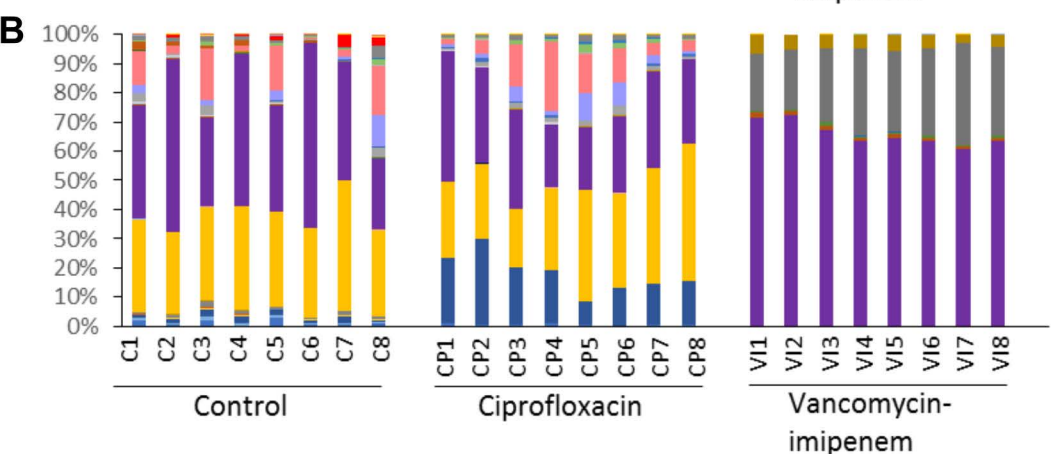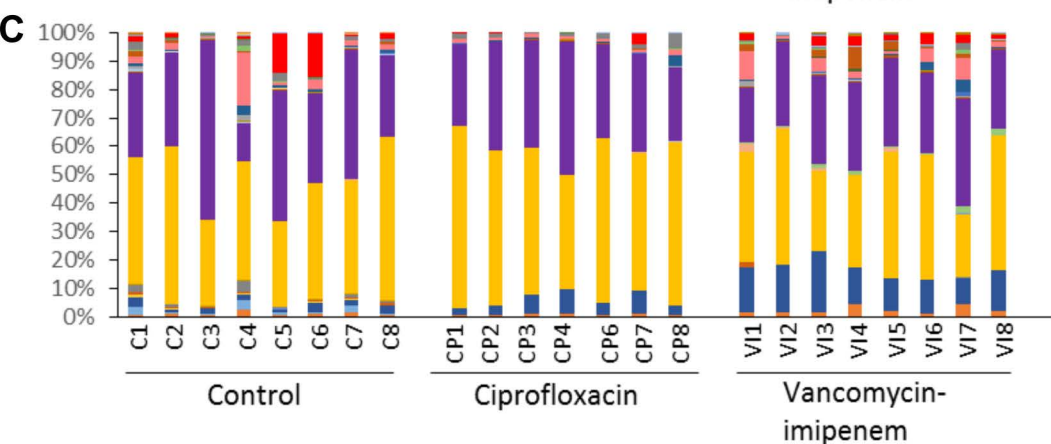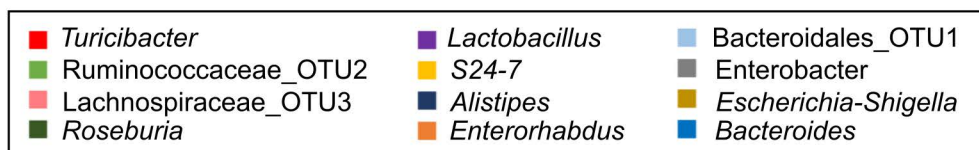

Supplement: FIG S1 [file sph001172231sf1.pdf]

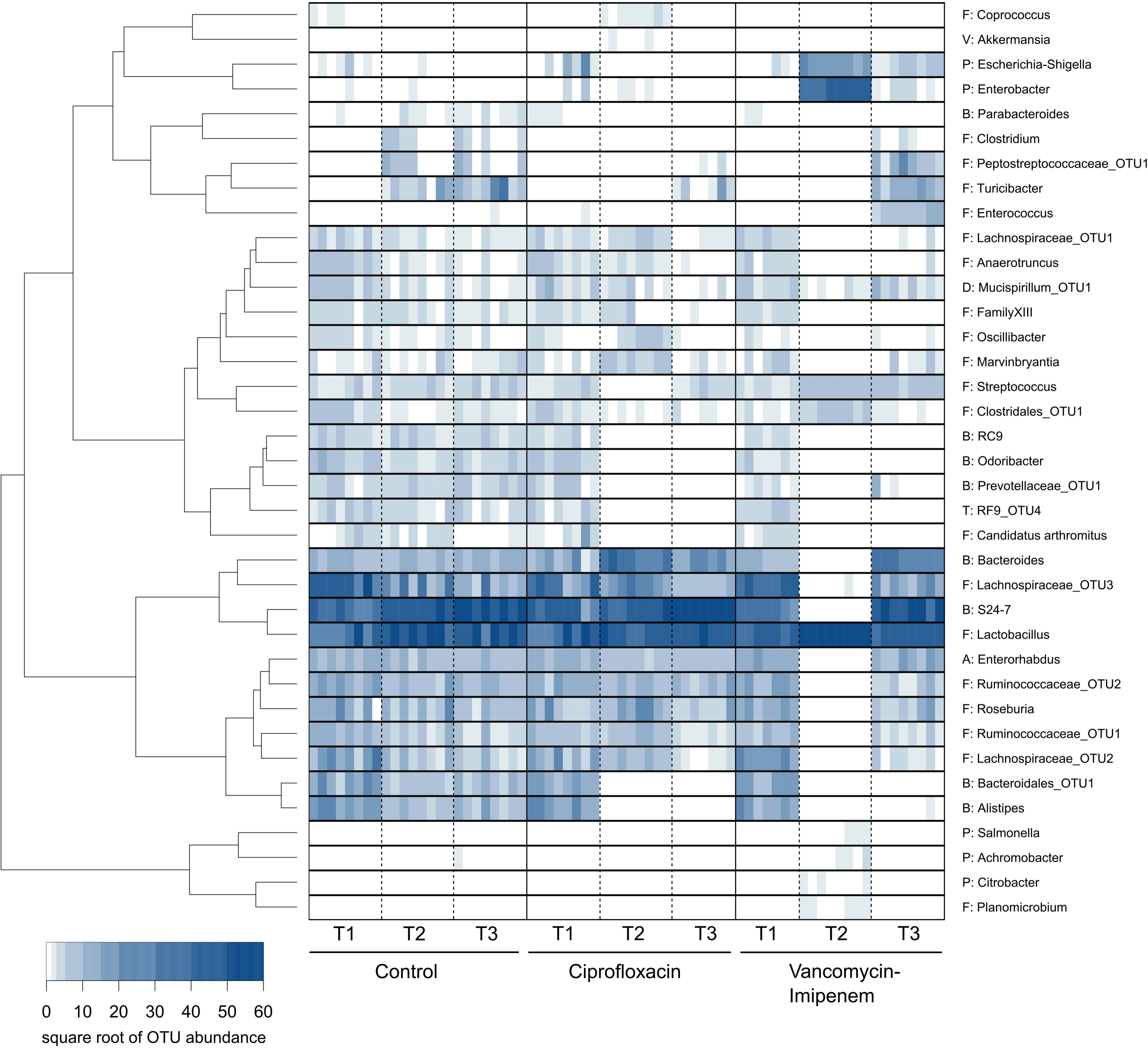

Supplement: FIG S2 [file sph001172231sf2.pdf]

**A**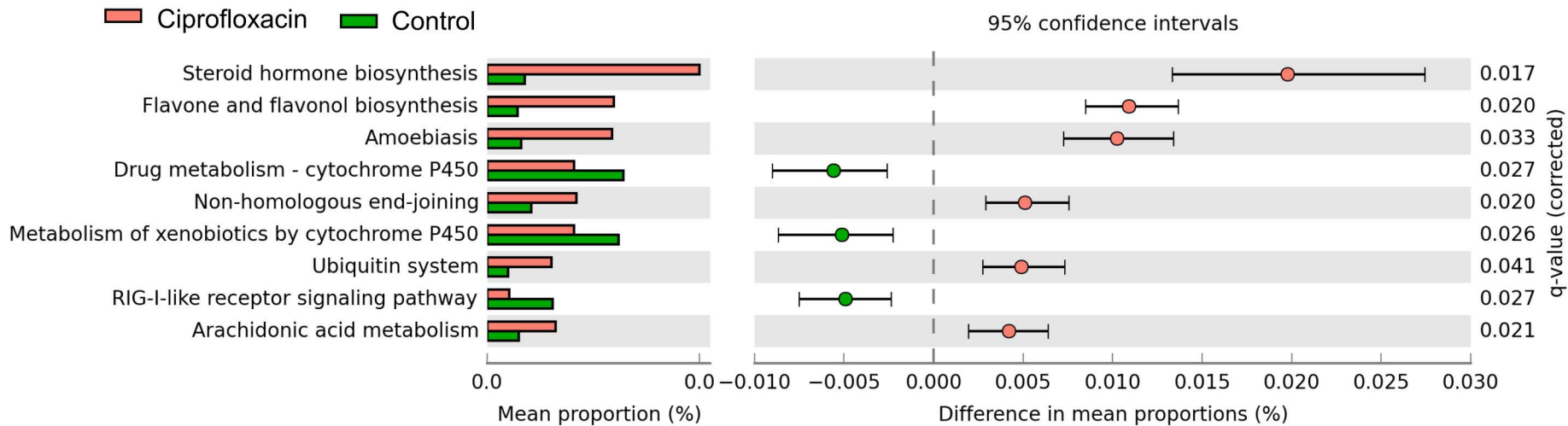**B**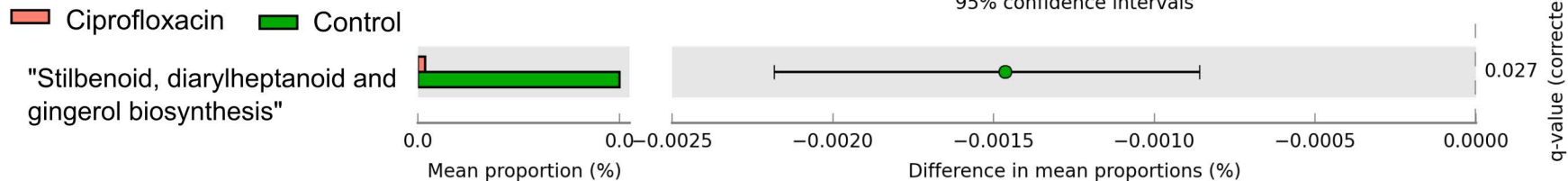

Supplement: FIG S3 [file sph001172231sf3.pdf]

**A**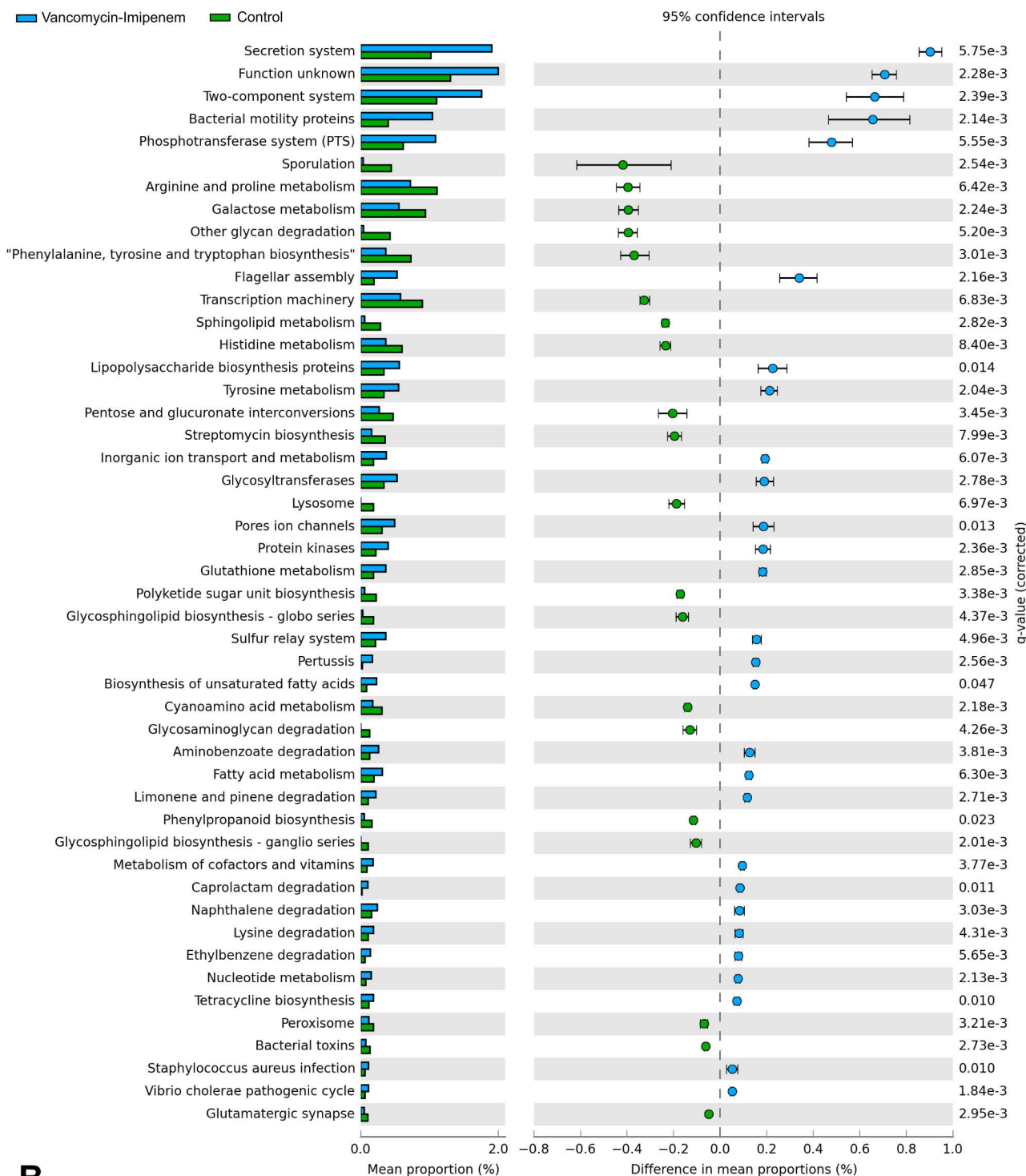**B**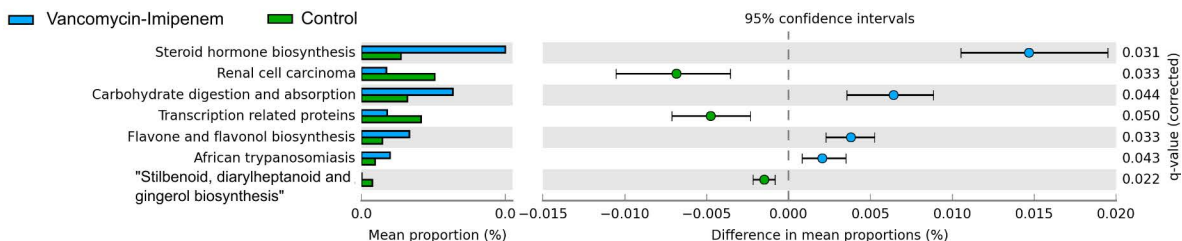

Supplement: FIG S4 [file sph001172231sf4.pdf]
