# Supplementary material for: Divergent Relationships between Fecal Microbiota and Metabolome following Distinct Antibiotic-Induced Disruptions
Source: mSphere. 2017 Feb 8;2(1):e00005-17. doi: 10.1128/mSphere.00005-17 (PMC5299068; doi:10.1128/mSphere.00005-17)
Supplement: TEXT S1 [file sph001172231s8.pdf]

Supplemental materials and methods

16S rRNA gene amplicon sequencing.

Amplicons of the V4 hypervariable region of the bacterial 16S rRNA gene were generated from DNA extracts, as described previously (62). Modified universal bacterial primer pairs 515F (5'-TCGTCGGCAGCGTCAGATGTGTATAAGAGACAGGTGCCAGCMGCCGCGGTAA-3') and 806R (5'-GTCTCGTGGGCTCGGAGATGTGTATAAGAGACAGGGACTACHVGGGTWTCTAAT-3'), with Illumina adapter overhang sequences (indicated by underline) were used for the amplification of the V4 hypervariable region of the bacterial 16S rRNA gene. The amplicons were generated from 25 PCR cycles, and indexes were attached to the amplicon with 8 PCR cycles. All amplicons were cleaned and sequenced according to the Illumina MiSeq 16S Metagenomic Sequencing Library Preparation protocol (http://support.illumina.com/downloads/16s_metagenomic_sequencing_library_preparation.html) with certain modifications. Specifically, PCR for amplicon generation was performed at a melting temperature of 50°C. Samples were multiplexed using a dual-index approach with the Nextera XT Index kit (Illumina, San Diego, USA) according to the manufacturer’s instructions. Amplicon library concentrations were determined using the Qubit HS dsDNA assay kit (Life Technologies, Melbourne, Australia). The final library was paired-end sequenced at 2 x 300 bp using a MiSeq Reagent Kit v3 on a Illumina MiSeq platform (Illumina, San Diego, USA), at the David R Gunn Genomics Facility, South Australian Health and Medical Research Institute.
